# Oral Microbiome Signatures in Indonesian Healthy, Gingivitis, and Periodontitis Subjects: A 16S rRNA Next-Generation Sequencing Analysis-Based Exploratory Study

**DOI:** 10.3390/dj14080532

**Published:** 2026-08-21

**Authors:** Benso Sulijaya, Melinda Rabekka Purba, Mardikacandra Manggala Putra, Fatimah Maria Tadjoedin

**Affiliations:** 1 Department of Periodontology, Faculty of Dentistry, Universitas Indonesia, Salemba Raya No. 4, Jakarta 10430, Indonesia; fatimah.tadjoedin@ui.ac.id; 2 Dental Division, Universitas Indonesia Hospital, Depok 16424, Indonesia; 3 Basic, Translational, and Clinical Research in Periodontology Group, Faculty of Dentistry, Universitas Indonesia, Jakarta 10430, Indonesia; 4 Periodontology Specialist Program, Department of Periodontology, Faculty of Dentistry, Universitas Indonesia, Jakarta 10430, Indonesia

**Keywords:** oral microbiome, periodontal status, gingivitis, periodontitis, NGS

## Abstract

**Background:** To date, several studies have confirmed the fact that over 700 species of microbiota interacts with host, modulating immunity, controlling the homeostasis environment, and thus maintaining systemic condition. Dysbiosis in the subgingival biofilm can initiate chronic inflammation of the gingiva, potentially progressing to periodontitis. Advances in DNA sequencing analysis of the subgingival microbial community have shown that periodontal treatment causes a microbial shift in subgingival plaque, affecting the taxonomic composition (disease- and health-associated taxa). Yet, none of these have been investigated in Indonesia. **Objective:** The objective was to profile the composition of oral microbiome in Indonesian population with healthy, gingivitis, and periodontitis status. Further, we analyzed the subgingival bacterial alteration following the therapy in periodontitis group. **Methods:** Twelve subjects consisting of healthy, gingivitis, and periodontitis patients were included. Additionally, the periodontitis group was observed at baseline, 1-month, and 3-month. Subgingival dental plaque were sampled and 16S rRNA NGS analysis was performed. Alpha (Chao1, Shannon, and Simpson indices) and beta diversity (PCoA plots based on Bray–Curtis dissimilarity) were observed. Microbiota composition at the genus and species levels was analyzed. **Results:** No statistically significant differences (*p* > 0.05) were found for Chao1, Shannon, and Simpson indices amongst groups and in periodontitis patients across the observation. At the genus level, PCoA plots based on Bray–Curtis dissimilarity revealed that the clinical status accounted for 21.9% of the total variation (R^2^ = 0.219, *p* = 0.197), while at the species level, it accounted for 19.8% (R^2^= 0.198, *p* = 0.281). Periodontitis samples across all timepoints showed no distinct clustering at either the genus 7.6% (R^2^ = 0.076 *p* = 0.097) or species levels 9.7% (R^2^ = 0.097 *p* = 0.995). Top five subgingival microbiota at the genus and species levels in all groups showed definite pattern of composition. Although no significant association was found (*p* > 0.05), it described that *Veillonella parvula*, *Campylobacter gracilis*, and *Capnocytophaga granulosa* were more abundant in healthy subjects than in gingivitis and periodontitis. *Prevotella oris* and *Selenomonas noxia* were less abundant in healthy subject than in gingivitis and periodontitis. In periodontitis subjects, *Prevotella intermedia* was increased by the therapy at 1 month and reduced again at 3 months. On the other hand, *Capnocytophaga granulosa* was increased by the therapy at 1 and 3 months. *Hoylesella loescheii* was reduced by the time. *Porphyromonas gingivalis* was reduced at 1 month and increased again at 3 months. **Discussion:** The result showed that the number of alpha diversity was notably higher in the gingivitis and periodontitis groups compared to health group, supporting a trend toward increased community richness and evenness in diseases states. The microbial richness and evenness remained relatively stable across the evaluated periods within periodontitis cohort. Oral microbial composition defines the periodontal status and disease. More abundance of Red Complex bacteria is associated with disease-associated condition. Pathogen re-colonization may occur 3 months after the therapy. **Conclusions:** These findings suggest that maintaining health-associated microbiome may be beneficial to the clinical status. Periodontal recall may be addressed from 1 to 3 months after the therapy to avoid bacterial re-colonization. Suppressing bacterial dysbiosis and regulating periodontal homeostasis are the main key in managing periodontal treatment.

## 1. Introduction

Periodontal disease is a chronic inflammatory condition that compromises the integrity of tooth-supporting structures, including the gingiva, periodontal ligament, and alveolar bone [1]. Pathogenesis of periodontal disease is mediated by the interaction of microorganisms in dental plaque biofilm and immune response of the host, and further influenced by local, environmental, and genetic aspects [2,3,4]. The current consensus shifts away from a simple bacterial infection model toward a dysbiotic shift in the oral microbiome [5]. The “Keystone Pathogen Hypothesis” suggests that *Porphyromonas gingivalis* manipulates the host immune system, turning a symbiotic microbial community into a destructive one [6].

For decades, the study of periodontitis was dominated by the search for a single causative agent. Early research identified the “Red Complex”—a triumvirate of *Porphyromonas gingivalis*, *Tannerella forsythia*, and *Treponema denticola*—as the primary drivers of tissue destruction [7]. However, contemporary research in 2026 suggests a more complex ecological model. The recent research has introduced the GF-MoR Complexes, which provide a more nuanced map of the 700+ species in the oral microbiome [8]. The Keystone Pathogen Hypothesis, *Porphyromonas gingivalis* (*P.gingivalis*), is now recognized as a “keystone” pathogen. Even in low abundance, *P.gingivalis* remodels a healthy microbial community into a disease-causing one by subverting the immune system (e.g., inhibiting IL-8 to prevent neutrophil recruitment) [9]. *Pg* utilizes gingipains (cysteine proteases) to degrade host proteins and sequester iron, which acts as a trigger for the dysbiotic community. By secreting specialized enzymes known as gingipains, it subverts the host’s innate immune system, turning a previously harmonious microbial community into a “dysbiotic” one [5]. This shift is not merely an increase in “bad” bacteria, but a total collapse of the oral ecosystem’s “eubiosis” (balance). In the end, the Red Complex (*P. gingivalis*, *Tannerella forsythia*, and *Treponema denticola*) represents the final, most destructive stage of biofilm maturation [5]. Moreover, the information regarding oral bacterial composition and assembly may develop effective and efficient approach for periodontal treatment [10].

Several studies found bacterial shifting in subgingival biofilm, including disease- and health-associated taxa composition and the bacterial quorum sensing in patient following periodontal therapy [11,12,13]. While Scaling and Root Planing (SRP) remains the foundation of periodontal treatment, adjunctive therapies are evolving. Host Modulation Therapy (HMT), specifically the use of sub-antimicrobial dose doxycycline, is used to inhibit matrix metalloproteinases (MMPs) that degrade the collagen matrix. Additionally, Antimicrobial Photodynamic Therapy (aPDT) is being explored as a non-invasive method to reduce bacterial load without contributing to antibiotic resistance [14].

While several studies have confirmed the interaction of over 700 species with the host, none of these transitions have been comprehensively investigated in Indonesia. Given the unique dietary and environmental factors of the Indonesian demographic, profiling these compositions is essential for precision periodontics. This study utilizes 16S rRNA Next-Generation Sequencing (NGS) to profile bacterial diversity and compositions in healthy, gingivitis and periodontitis subjects, and further to monitor the efficacy of SRP over a 3-month longitudinal period in periodontitis subject.

## 2. Materials and Methods

### 2.1. Study Design and Subject Recruitment

The study protocol was approved by the Dental Research Ethics Committee, Faculty of Dentistry, Universitas Indonesia, letter number: 01/Ethical Approval/FKGUI/I/2022 and protocol number: 091241221. This study included 12 Indonesian subjects categorized into three groups: healthy (4), gingivitis (4), and periodontitis (4). Inclusion criteria were (1) female patients aged 25–60 years; (2) diagnosed as health, gingivitis, and periodontitis in accordance with the American Academy of Periodontology (AAP) and the European Federation of Periodontology (EFP) periodontal classification system [1]. Further, the periodontitis group was observed at baseline, 1-month, and 3-month intervals following SRP therapy. The exclusion criteria were as follows: (1) systemic diseases that might affect the study (such as diabetes mellitus, cardiovascular diseases, blood disorders, etc.) [15,16,17,18]; (2) currently taking medication especially for antibiotics [16,19,20]; (3) current smokers [16,21]; (4) pregnancy [16,21,22]; and 50 and (5) orthodontic appliances or extensive prosthodontic restorations. To minimize confounding systemic variations and maximize community homogeneity within this pilot cohort, subject selection was restricted exclusively to female participants. Potential participants who were pregnant, lactating, or taking hormonal contraceptives were excluded to avoid acute endocrine-driven shifts in the subgingival microenvironment.

### 2.2. Periodontal Status

Clinical periodontal parameters, including probing depth (PD), clinical attachment loss (CAL), and bleeding on probing (BOP), were recorded full-mouth at six sites per tooth by a calibrated examiner (M.R.B and M.M.P). Diagnostic categorization strictly followed the 2017 AAP/EFP authoritative classification: Periodontally Healthy: PD ≤ 3 mm, no CAL, and BOP < 10%; Gingivitis: PD ≤ 3 mm, no CAL, and BOP ≥ 10%; Periodontitis: Presence of interdental CAL at ≥2 non-adjacent teeth, with PD ≥ 4 mm [1].

### 2.3. Sampling Site Selection

Subgingival dental plaque was sampled from all subjects. Further, in periodontitis subjects, subgingival plaque was re-collected from the same site at one month and three months after the SRP done at the baseline. To collect subgingival samples, the tooth was isolated using a cotton roll, subgingival biofilm was collected using sterile standard #30 paper points inserted into the pocket for 30 s. Samples were placed immediately into a sterile Eppendorf tube containing 1000 μL of phosphate-buffered saline and labeled. Samples were immediately transferred to a portable cooler box at 4 °C, transported to the laboratory within 2 h of collection, and stored at −80 °C until DNA extraction.

### 2.4. Molecular Analysis (DNA Extraction and Sequencing)

Microbial genomic DNA was extracted from subgingival plaque samples using the DNA Extraction Kit (InstaGeneTM Matrix, Bio-Rad, Hercules, CA, USA). DNA concentration was measured using both NanoDrop spectrophotometers and Qubit fluorometers (Thermo Fisher Scientific, Waltham, MA, USA). Library preparation and sequencing was done using NGS platform, Nanopore sequencing (full-length sequence of 16s rRNA gene [V1–V9 regions]) (Oxford Nanopore Technology, Oxford, UK) [23]. Nanopore sequencing was generated by MinKNOW software version 23.04.5 (Oxford Nanopore Technology, Oxford, UK). Base-calling was performed using Guppy version 6.5.7 (Oxford Nanopore Technology, Oxford, UK) with a high-accuracy model [24]. Amplification was executed using the universal primer pair 27F (5′-AGAGTTTGATCMTGGCTCAG-3′) and 1492R (5′-TACGGYTACCTTGTTACGACTT-3′). The PCR program consisted of an initial denaturation at 95 °C for 3 min, followed by 30 cycles of 95 °C for 30 s, 55 °C for 30 s, and 72 °C for 1 min, with a final extension at 72 °C for 5 min. High-throughput sequencing was performed utilizing the Oxford Nanopore platform (v.2.8.0) to achieve deep taxonomic assignment.

### 2.5. Quality Filtering and Pre-Processing

Initial quality control was performed using NanoPlot to assess read length distribution and mean quality scores. The quality of FASTQ files was visualized using NanoPlot, and quality filtering was performed using NanoFilt (v.2.8.0) (Oxford Nanopore Technology, Oxford, UK) [25,26]. We applied a minimum read length threshold of 1200 bp and a minimum quality score (Q-score) of >10. Filtered reads were classified using a centrifuge classifier. Filtered reads were aligned for taxonomic classification using the Centrifuge engine. The reference database used was the Human Oral Microbiome Database (HOMD) v15.22, which provides species-level resolution for over 700 oral taxa. A confidence threshold of 0.8 was applied to ensure the accuracy of species-level assignments. Any reads falling below this threshold or failing to align to the HOMD were categorized as “Unclassified.” The bacteria index was built using the NCBI 16S RefSeq database (https://ftp.ncbi.nlm.nih.gov/refseq/TargetedLoci/ (Accessed on 1 August 2025)). Downstream analysis and visualizations were performed using Pavian (http://github.com/fbreitwieser/pavian (Accessed on 1 August 2025)) [27], Krona Tools (https://github.com/marbl/Krona/wiki/KronaTools (Accessed on 1 August 2025)) [28], and R version 4.2.3 [29].

### 2.6. Contamination Control and Data Integrity

To address potential environmental or laboratory contamination, “decontam” protocols were followed by comparing sample profiles against negative extraction and sequencing controls. Taxa identified as potential contaminants (e.g., those appearing with higher frequency in negative controls than in clinical samples) were pruned from the final dataset.

### 2.7. Statistical Analysis and Sample Size Calculation

Due to the exploratory and pilot nature of this study, a formal power calculation was not performed. Data were then analyzed using SPSS software version 26 (IBM, Chicago, IL, USA), and the visualization data were made with GraphPad Prism software version 10.1.0 (GraphPad, Boston, MA, USA). Alpha diversity and microbial composition data for cross-sectional and longitudinal data were analyzed with the Kruskall–Wallis and Friedman, respectively. Permutational Multivariate Analysis of Variance (PERMANOVA) was utilized to test the significance of community clustering based on clinical status and timepoints. The coefficient of determination (R^2^) was reported to quantify the percentage of microbial variance explained by the clinical group or observation period. For all statistical tests, a *p*-value < 0.05 was considered the threshold for formal statistical significance. In cases where *p*-values exceeded this threshold, but effect sizes were large, the results were interpreted as indicating biological trends or marginal ecological transitions.

## 3. Result

**Figure 1:**
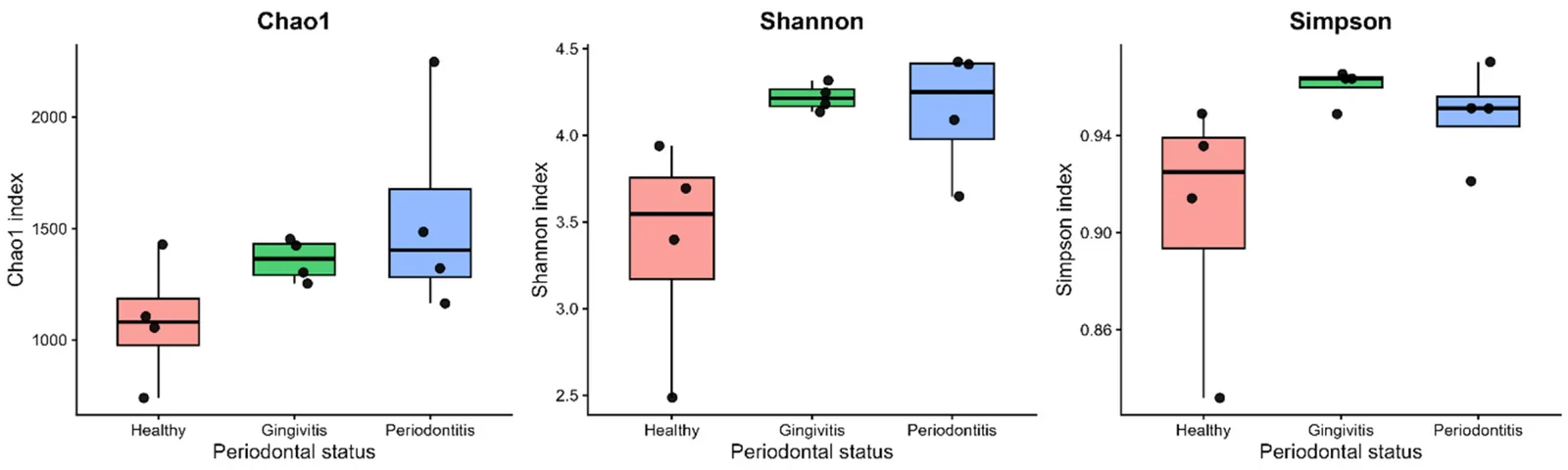
Comparison of alpha diversity between the health, gingivitis, and periodontitis groups.

**Figure 2:**
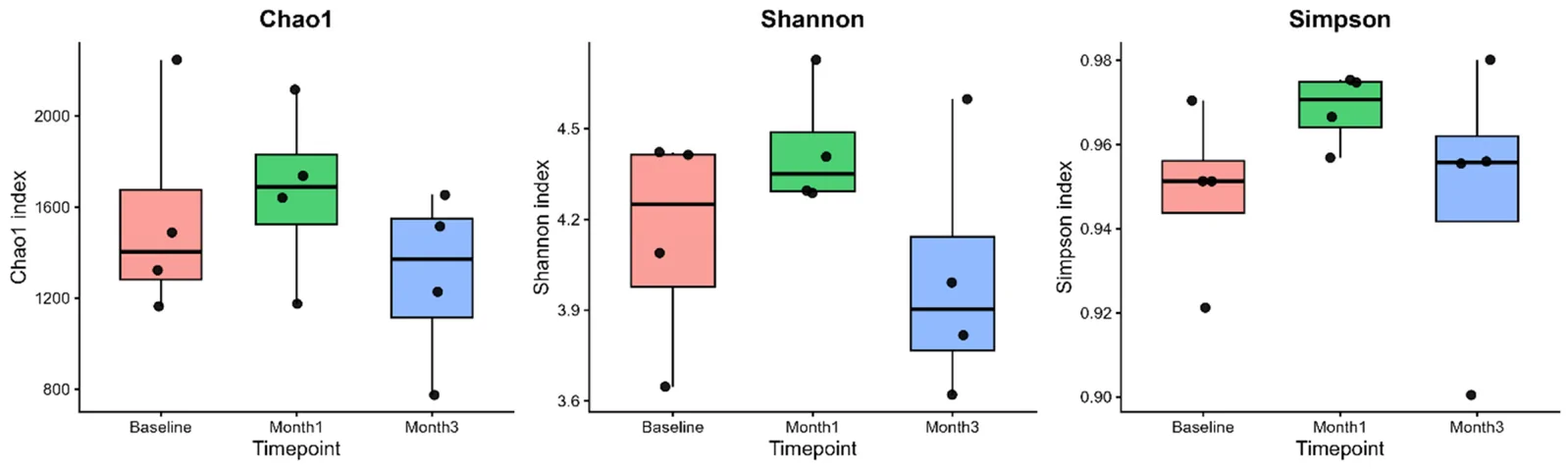
Alpha diversity between baseline, 1-month, and 3-month visits in the periodontitis group.

Subgingival microbiota diversity was evaluated using the Chao1, Shannon, and Simpson indices. As shown in Figure 1, the number of these three indices were notably higher in the gingivitis and periodontitis groups compared to health group, indicating a trend toward increased community richness and evenness in diseases states. However, these differences were not statistically significant (*p* > 0.05) (Table 1). Despite this, the effect size analysis yielded a large magnitude (η^2^ >0.14) for all alpha diversity indices. In addition, the longitudinal changes in subgingival microbial diversity of periodontitis subjects were analyzed using the Friedman test (Figure 2). The result showed no statistically significant differences (*p* > 0.05) in Chao1, Shannon, and Simpson indices across the observation. Furthermore, the Kendall’s W coefficient indicated a small effect size, suggesting that the microbial richness and evenness remained relatively stable across the evaluated periods within this cohort (Table 2).

**Figure 3:**
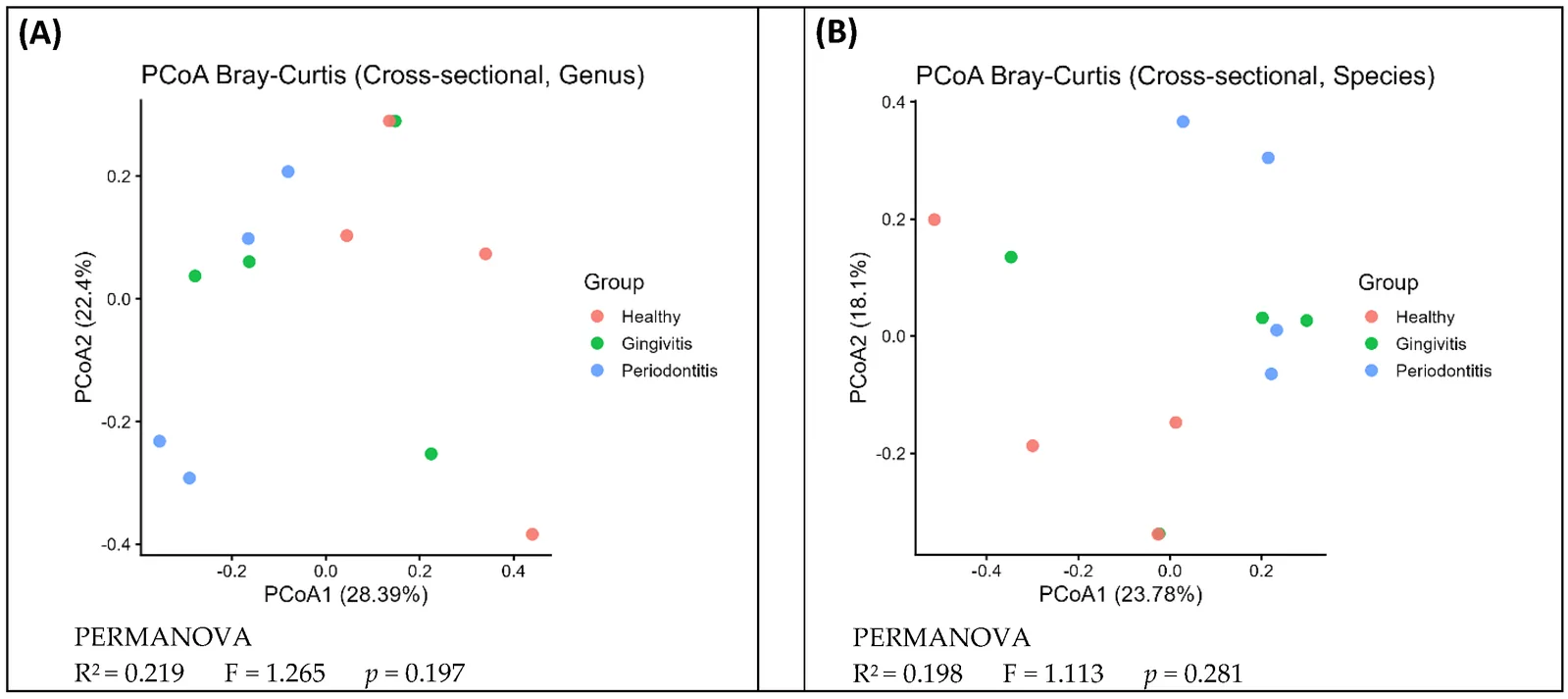
Beta diversity analysis of subgingival microbiota in the health, gingivitis, and periodontitis groups. Principal Coordinates Analysis (PCoA) plots based on Bray–Curtis dissimilarity showing the microbial community structure at the (**A**) genus and (**B**) species level. (**A**) **Genus** clustering shifts correspond to a decline in *Capnocytophaga* and an enrichment of *Prevotella* from health to periodontitis. (**B**) **Species** distribution is primarily characterized by the depletion of the health-associated *Capnocytophaga granulosa* baseline and the proliferation of disease-associated *Selenomonas noxia* and *Prevotella oris* signatures.

**Figure 4:**
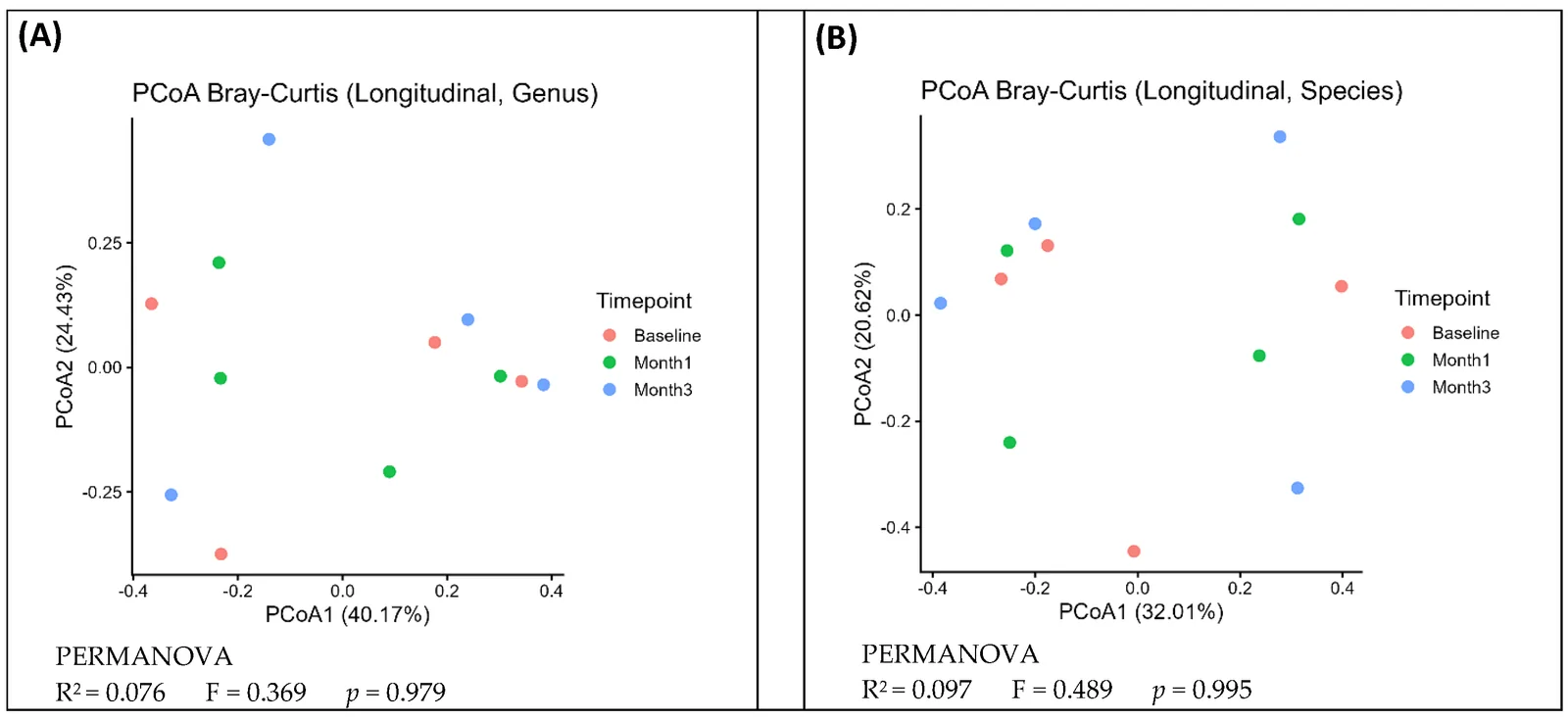
Beta diversity analysis of subgingival microbiota between baseline, 1-month, and 3-month visits in the periodontitis groups. Principal Coordinates Analysis (PCoA) plots based on Bray–Curtis dissimilarity showing the microbial community structure at the (**A**) genus and (**B**) species level. This spatial continuity highlights a high level of microbial resilience, supported by the stable presence of *Porphyromonas gingivalis* and a post-therapy pathogenic rebound observed at the 3-month threshold.

As shown in Figure 3, beta diversity was visualized using PCoA plots based on Bray–Curtis dissimilarity at both the genus (Figure 3A) and species levels (Figure 3B). At the genus level, the clinical status accounted for 21.9% of the total variation (R^2^ = 0.219, *p* = 0.197), while at the species level, it accounted for 19.8% (R^2^ = 0.198, *p* = 0.281). Although the plots show a high degree of overlap among the healthy, gingivitis, and periodontitis groups, the PERMANOVA results indicate that the global microbial community structure did not differ significantly across the three conditions (*p* > 0.05). In the Figure 3A, genus clustering shifts correspond to a decline in *Capnocytophaga* and an enrichment of *Prevotella* from health to periodontitis. Moreover, species distribution is primarily characterized by the depletion of the health-associated *Capnocytophaga granulosa* baseline and the proliferation of disease-associated *Selenomonas noxia* and *Prevotella oris* signatures (Figure 3B). Further, longitudinal beta diversity analysis was conducted in periodontitis to assess the stability of the subgingival microbiota at baseline, one month, and three months post-intervention (Figure 4). PCoA plots based on Bray–Curtis dissimilarity revealed that samples across all timepoints remained largely intermingled, with no distinct clustering observed at either the genus (Figure 4A) or species levels (Figure 4B). At the genus level, PERMANOVA analysis indicated that periodontal status per timepoints accounted for 7.6% of the microbial variance (R^2^ = 0.076 *p* = 0.979). Although this difference did not reach the formal threshold for statistical significance, a marginal trend toward community separation was noted 9.7% of the microbial variance (R^2^ = 0.097 *p* = 0.995). This spatial continuity highlights a high level of microbial resilience, supported by the stable presence of *Porphyromonas gingivalis* and a post-therapy pathogenic rebound observed at the 3-month threshold. This suggests that the subgingival microbiome begins to undergo an ecological transition as periodontal conditions shift, even if the global community structure has not yet reached a state of distinct separation.

**Figure 5:**
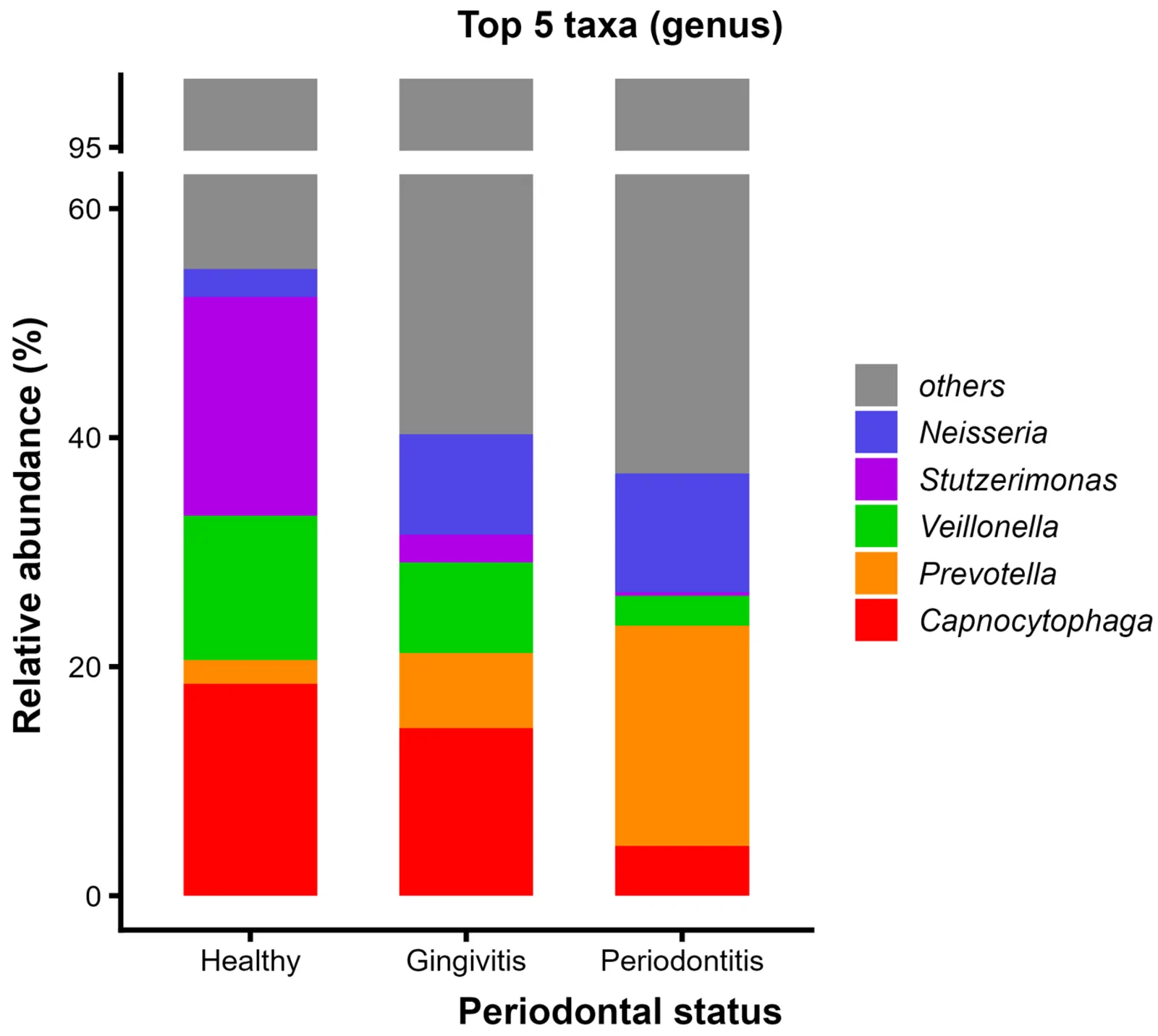
Composition of subgingival microbiota at the genus level in health, gingivitis, and periodontitis subjects.

**Figure 6:**
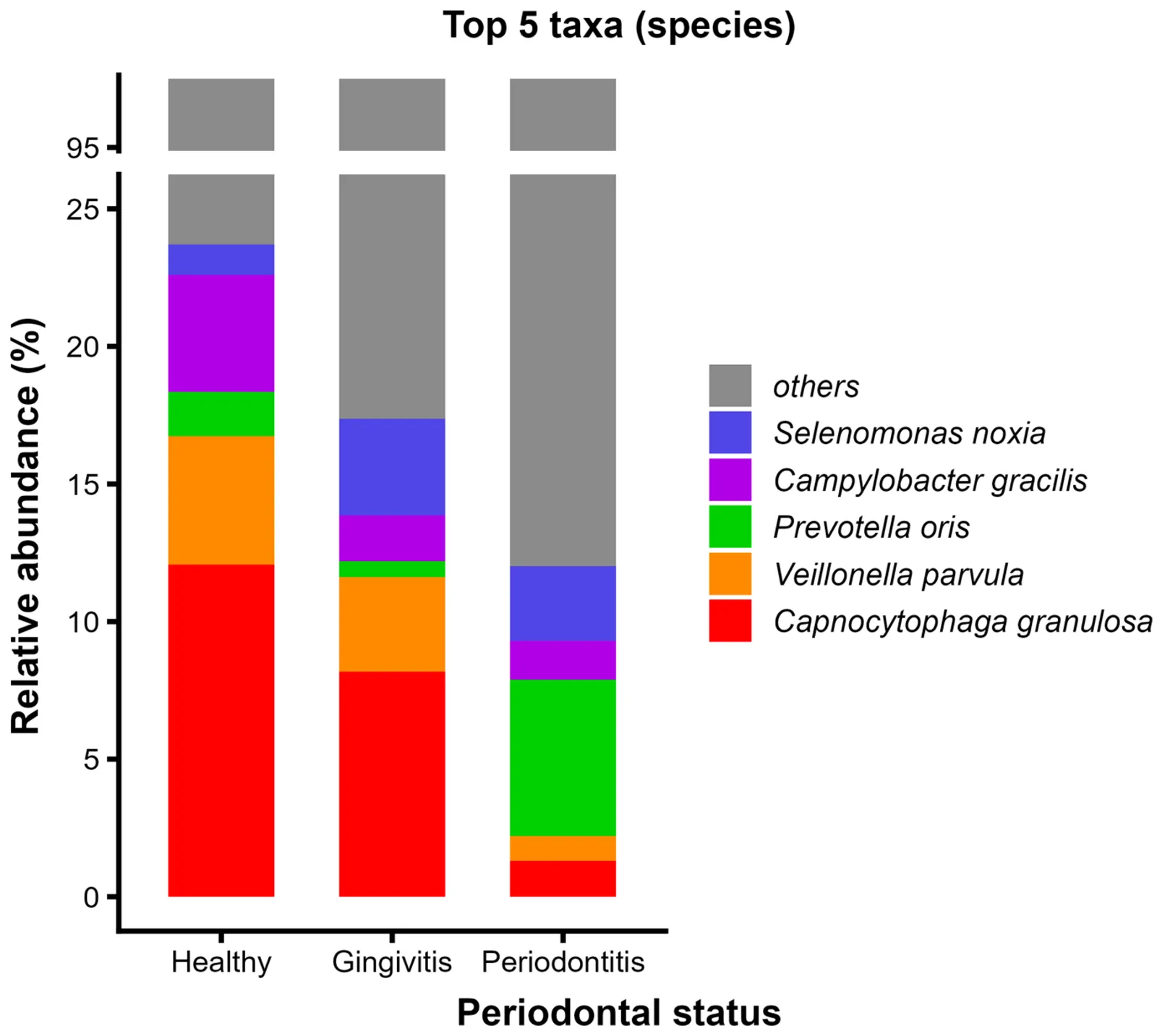
Composition of subgingival microbiota at the species level in health, gingivitis, and periodontitis subjects.

Figure 5 and Figure 6 demonstrate a profound taxonomic shift in the oral microbiome as periodontal health deteriorates from a healthy state to gingivitis and, ultimately, periodontitis. The data demonstrate a clear transition from a community dominated by commensal species to one characterized by pathogenic dysbiosis. As seen, the commensal dominance in health subject. In the healthy state, the oral microbiome is characterized by a high relative abundance of commensal bacteria. *Capnocytophaga* (specifically *C. granulosa*) and *Veillonella* (*V. parvula*) constitute the vast majority of the identified microbial population. In healthy samples, these species occupy roughly 25% of the total microbial community. Notably, classic periodontopathogens like *Prevotella was* virtually absent or exist at negligible levels. In contrast to gingivitis, there is a transitional shift in gingivitis that marks the initial breakdown of this stable commensal population. As commensal decline, there is a visible reduction in the relative abundance of *Campylobacter gracilis.*

This study found an emerging increase in *Prevotella oris* and the Gram-negative crescent-shaped pathogen, specifically *Selenomonas noxia* disease-associated condition. The “Others” category in gingivitis begins to expand, suggesting that the thinning of dominant commensal species allows for a more diverse, opportunistic microbial environment. In the periodontitis group, the microbiome undergoes a near-complete taxonomic reversal. The identified pathogenic cluster—comprising *Prevotella*—reaches its peak relative abundance 12.18% in periodontitis group. Species associated with health, such as *Capnocytophaga* and *Veillonella*, are reduced to minimal levels (less than 5–10% combined). Most strikingly in periodontitis group, the “Others” category expands to dominate the profile (exceeding 60% relative abundance). This indicates that advanced disease is characterized by a highly complex, heterogeneous, and disorganized microbial community that lacks the stable signature of a healthy oral cavity.

A marked increase was observed in species traditionally associated with the “Orange Complex” of periodontal pathogens (Table 3 and Table 4). Regarding *Prevotella oris* and *Selenomonas noxia*, these species were nearly undetectable in healthy subjects (0.18% and 0.60%). However, both species showed a substantial increase in subjects with gingivitis (2.12% and 0.19%), respectively) and maintained elevated levels in periodontitis (0.26% and 2.55%). *Prevotella oris* demonstrated its highest prevalence during the periodontitis stage (0.26%), representing a significant jump from its total absence in the healthy group (0.18%). *Capnocytophaga* in the healthy was a dominant genus (18.63%). This prevalence dropped to 8.88% in gingivitis and further marked to 4.17% in periodontitis. *Capnocytophaga granulosa* specifically followed this trend, decreasing from 11.02% in healthy group to 1.37% in periodontitis. *Veillonella parvula*, known as a common early colonizer in healthy oral cavity, decreased from 0.20 (4.72)% in the healthy group to a negligible 1.00 (0.82)% in the periodontitis group. *Campylobacter gracilis* showed a moderate presence in healthy subjects (4.56%) but decreased by approximately 50% in the periodontitis group (1.32%). Several other unnamed species (identified by genus in Table 3) mirrored the decline of the *Capnocytophaga*, with one genus falling from 18.63% in healthy group to 4.17% in the periodontitis group.

**Figure 7:**
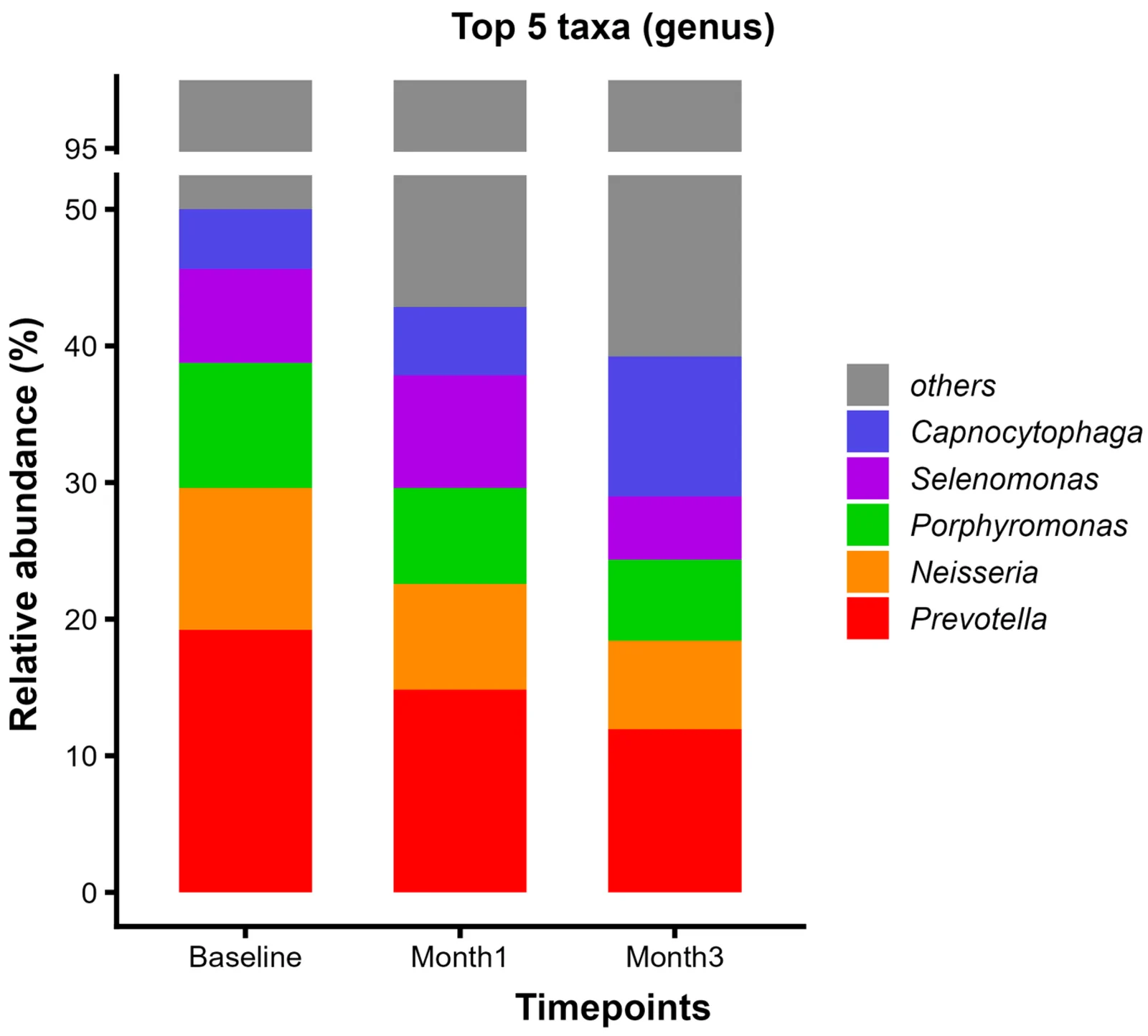
Composition of subgingival microbiota at the genus level in periodontitis (baseline, 1-month, and 3-month).

**Figure 8:**
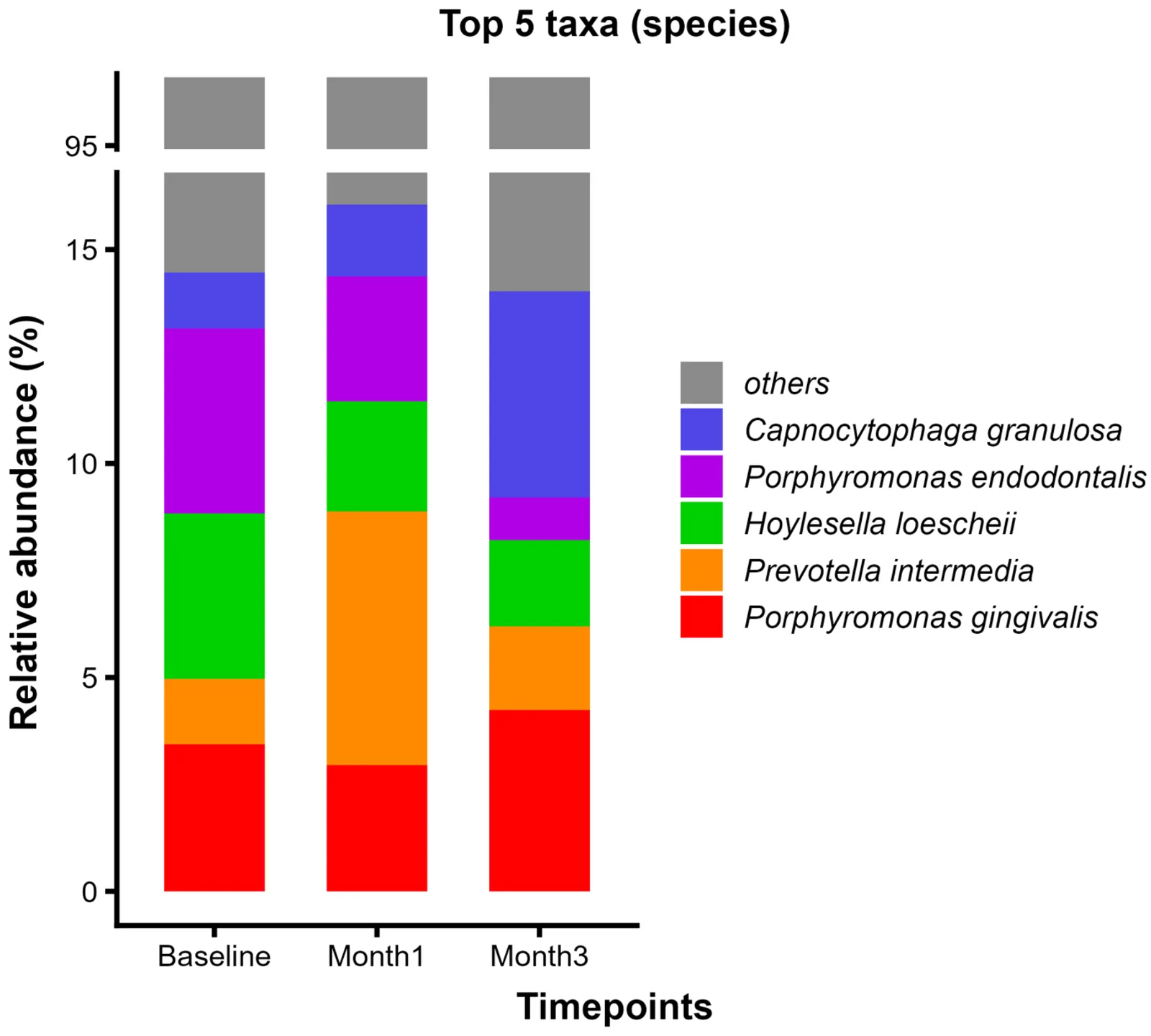
Composition of subgingival microbiota at the species level in periodontitis (baseline, 1-month, and 3-month).

Figure 7 and Figure 8 show a longitudinal changes in subgingival microbiota based on genus- and species-level relative abundance. At the baseline, the subgingival environment was characterized by a high relative abundance of genera typically associated with chronic periodontitis. The *Porphyromonas* and *Prevotella* represented a combined relative abundance of approximately 30%. Then, a significant portion of the community (nearly 65% at the species level) was categorized as “Others,” indicating a diverse but dysbiotic baseline state. One month following periodontal therapy, the microbial profile underwent a distinct shift, likely reflecting the mechanical disruption of the biofilm. At genus level, there was a notable increase in the relative abundance of *Capnocytophaga* and *Veillonella*. This study observed a transient shift at the species level; *Prevotella intermedia* showed a temporary spike in relative abundance, while *Porphyromonas gingivalis* levels began to decline compared to baseline. The proportion of “Other” species decreased significantly, as specific colonizers began to dominate the recovering niche. By the 3-month mark, the microbiota trended toward a new equilibrium characterized by an increase in health-associated commensals. The genus *Capnocytophaga*—specifically the species *Capnocytophaga granulosa*—maintained a significantly higher relative abundance than at baseline. While the genus *Porphyromonas* appeared to return toward baseline volumes, the species-specific data reveals that *Porphyromonas gingivalis* remained suppressed relative to its initial state. *Hoylesella loescheii* established a stable presence, contributing to a more balanced microbial profile compared to the pre-treatment phase.

At the genus level of periodontitis subjects, the microbial profile was characterized by a high degree of variability (Table 5). *Prevotella* remained the most dominant genus throughout the study, starting at a baseline of 12.18 (24.02)% and concluding at 2.74 (10.61)% at three months. *Porphyromonas* showed a slight initial decrease at one month (6.58 (11.36)%) before returning to near-baseline levels by the third month (6.33 (6.03)%). Conversely, *Capnocytophaga*, exhibited a gradual upward trend in their mean values over the 3-month period. Despite these fluctuations, all associated *p*-values remained > 0.05 (ranging from 0.174 to 1.0), indicating that the therapy did not induce a statistically significant shift in the overall genus composition. As shown in the Table 6, the species-specific data mirrors the trends observed at the genus level, showing stability in the presence of key periodontal pathogens. *Porphyromonas gingivalis*, a primary pathogen in periodontitis, remained relatively stable, with a baseline of 2.63 (5.41)% and a 3-month value of 3.50 (7.00)%. *Prevotella intermedia* showed a notable numerical spike at 1 month 3.48 (9.13)% compared to baseline 1.29 (1.18)%, but this concentration levelled off significantly by the three-month mark 1.30 (1.81)%. Other species, including *Capnocytophaga granulosa* and *Hoylesella loescheii*, showed modest increases in their mean counts over time.

## 4. Discussion

The present study shows taxonomic shift in the oral microbiome as periodontal health deteriorates from a healthy state to gingivitis and, ultimately, periodontitis in Indonesia. A notable characteristic of this study is the exclusive inclusion of female participants. While this selective criteria effectively eliminated sex-dependent immune profiles and hormonal variances as confounding factors in a small sample size, it restricts the external validity of our data. Given that sexual dimorphism significantly modulates both host immune reactivity and subgingival microbial colonization, future expanded cohort studies must include diverse male and female populations to validate these microbial patterns across broader demographics.

The analysis of subgingival microbial communities reveals an intriguing ecological narrative. In the cross-sectional comparison, alpha diversity indices (Chao1, Shannon, and Simpson) were notably higher in the gingivitis and periodontitis groups compared to the healthy cohort. While these differences did not reach formal statistical significance (*p* > 0.05), the effect size analysis yielded a large magnitude (η^2^ > 0.14) across all indices.

To ensure data reliability given the cohort size, diversity evaluations were interpreted using both non-parametric hypothesis testing and variance/effect size metrics. The large effect sizes (η^2^ > 0.14) observed in cross-sectional alpha diversity indices highlight important biological trends in community richness despite the lack of formal *p*-value significance. Furthermore, the utilization of PERMANOVA allowed us to reliably quantify that clinical status dictates roughly 20% of the subgingival microbial variance (R^2^ = 0.219 for genus, R^2^ = 0.198 for species), validating the ecological weight of the clinical classification over random baseline noise. This suggests that while the sample size may limit statistical power, there is a substantial biological trend toward increased community richness as periodontal health declines. This finding aligns with established models where periodontitis is uniquely characterized by increased bacterial diversity and richness rather than a loss of diversity [30]. The presentation of quantitative effect sizes in addition to *p*-values is increasingly recognized as a vital step in evaluating biologically relevant effects that may otherwise be obscured by statistical constraints [31,32].

Beta diversity analysis further supports this shifting ecological state. At both the genus and species levels, clinical status accounted for approximately 19.8%–21.9% of the total microbial variation. Despite the lack of distinct clustering in the PCoA plots and non-significant PERMANOVA results (*p* > 0.05), the variance explained (R^2^) indicates that clinical status is a meaningful driver of community composition. This is consistent with recent findings that clinical status, alongside other biological factors, significantly influences the overall subgingival microbiome composition [33]. Longitudinally, the subgingival microbiota demonstrated remarkable stability over the 3-month observation period. The Friedman test revealed no significant shifts in alpha diversity, and Kendall’s W indicated only a small effect size, suggesting that the richness and evenness of the community remained relatively constant post-baseline. The longitudinal beta diversity results showed significant intermingling of samples across all timepoints, with a very low variance explained by time (R^2^ from 7.6% to 9.7%). This lack of distinct separation suggests a high level of microbial resilience within this cohort. Such resilience is often observed in subgingival niches where the global community structure does not reach a state of complete “regime shift” without significant environmental perturbations. Recent intervention studies have similarly noted high ecological resilience, where even therapeutic or adjunct measures failed to induce significant microbiome changes over a three-month period despite clinical improvements [34]. The marginal trends observed—particularly the large effect sizes in cross-sectional richness—may indicate that the subgingival microbiome begins to undergo ecological transitions early in the disease process. However, the global community structure remains stable within the 3-month window, underscoring the necessity of long-term monitoring to determine if these subtle transitions eventually manifest as significant taxonomic shifts.

In terms of microbial composition, our findings demonstrate a clear transition from a community dominated by commensal species to one characterized by pathogenic dysbiosis. Moreover, the results indicate that while periodontal therapy provides an immediate taxonomic shift, the microbial community exhibits a tendency toward re-colonization by 3 months. This indicates that oral microbial composition is a potential marker of periodontal status. The rapid re-colonization observed at 3 months suggests that mechanical therapy provides only a temporary “ecological reset”.

*Veillonella parvula* and *Capnocytophaga granulosa* were identified as health-associated taxa, showing significantly higher abundance in the healthy group compared to gingivitis and periodontitis. Our study in line with previous finding that *Veillonella* and *Capnocytophaga* are significantly more abundant in periodontally healthy individuals compared to those with gingivitis or periodontitis [35]. In healthy states, the dominance of *Capnocytophaga* and *Veillonella* acts as a biological shield to prevent the colonization of “Red Complex” pathogens (like *P. gingivalis*) through competitive exclusion and local environment stabilization [35]. Further, *Veillonella parvula* is typically associated with homeostatic conditions and helps maintain a neutral pH by converting lactic acid (produced by cariogenic bacteria) into weaker acids, which helps maintain the biofilm pH above the critical threshold for enamel demineralization [36], thus preventing the colonization of more aggressive species.

As the oral environment shifts toward disease (gingivitis and periodontitis), the microbial composition changes significantly. Species such as *Prevotella intermedia* and *Selenomonas noxia* show an increase in abundance in diseased subjects compared to healthy ones. *Selenomonas noxia*, a member of the Gram-negative crescent-shaped pathogen of periodontal pathogens, was detected exclusively in the diseased Indonesian subjects studied. A previous study by Ningsih et al. found that the number of microbiome population in gingivitis patients had no significant different than those with periodontitis (0.445 × 10^6^ CFU/mL vs. 0.047 × 10^6^ CFU/mL), *p* = 0.306 [37]. In addition to this, the increasing severity, extent, and grade of periodontitis were associated with enrichment of established periodontitis-related genera (e.g., *Dialister*, *Filifactor*, *Fusobacterium*, *Porphyromonas*, *Prevotella*, *Tannerella*) and depletion of health-related genera (e.g., *Rothia*, *Veillonella*). Those genera were altered in Stage III–IV periodontitis, relative to participants with no or localized Stage I–II periodontitis [38]. However, the presence of these specific bacteria serves as a signature for the transition from a healthy state to a pathological one.

The research also tracked microbial changes in periodontitis subjects following treatment of SRP at 1-month and 3-month intervals. At 1 month, SRP successfully reduced levels of *P. intermedia* and *P. gingivalis* while simultaneously increasing the presence of health-associated *C. granulosa*. However, at the 3-month interval, a resurgence of pathogens was observed; levels of *P. intermedia* and *P. gingivalis* began to rise again, while *C. granulosa* levels declined. This phenomena known as “U-Shaped” recovery or bacterial rebound. While non-surgical therapy SRP effectively reduces pathogen loads and increases commensal levels within one month, a “U-shaped” recovery often occurs by three months, where pathogen levels begin to return to baseline [39]. This highlights the necessity of supportive periodontal maintenance visits to restore microbial homeostasis.

Our finding shows the spike in *Selenomonas noxia* and *Prevotella oris* during the gingivitis phase suggests these species act as “bridge” organisms. They alter the local environment often by increasing inflammation and lowering oxygen levels to pave the way for the more destructive anaerobic pathogens seen in advanced periodontitis. A recent longitudinal study using high-resolution sequencing show that the microbiome changes asynchronously after cleaning [40]. Specific species like *P. intermedia* and *C. gracilis* act as markers of “dysbiotic turning points,” where their overgrowth precedes the irreversible tissue destruction characteristic of advanced periodontitis [41]. In early-to-moderate periodontitis, novel species such as *Fretibacterium* spp. and *Saccharibacteria* spp. (formerly TM7) are highly prevalent, acting alongside the orange complex to drive the transition toward deep pocket formation [42]. While *Fusobacterium nucleatum* is the primary physical bridge, *P. intermedia* has been identified as a dominant species in the progression of both periodontitis and peri-implantitis, contributing significantly to the proteolytic environment of the pocket [41,43].

Inflammation may also change the environment (e.g., increasing GCF flow and protein availability), which specifically favors the overgrowth of *P. intermedia* and *C. gracilis*. These “pathobionts” then manipulate the immune system to allow the Red Complex to colonize [44]. Contemporary research shifts focus on the Keystone Pathogen Hypothesis rather than just building a physical bridge; organisms like *P. intermedia* thrive on the inflammatory byproducts (like heme from bleeding) created by the host’s immune response. This creates a “feedback loop” that selects for these specific anaerobic pathogens [44,45,46]. Bring this to the clinical setting, traditional 6-month recall intervals may be insufficient for the Indonesian periodontitis demographic. Because pathogen re-colonization begins to manifest significantly between months 1 and 3, a 3-month maintenance schedule is necessary to disrupt the emerging Red Complex bacteria. Clinicians should utilize the 1-month post-therapy mark as the “biological baseline” to evaluate patient response. Moreover, beyond mechanical SRP, the use of quorum quenching agents or host modulation may be necessary to address the molecular drivers of the 3-month relapse.

The limitation of this study was likely attributed to the high standard deviations observed across all samples, indicating substantial inter-individual variability in the microbial composition of the subgingival plaque within each clinical category. Our participant selection was strictly limited to a single geographic cohort of female subjects, omitting potential sex-specific ecological variations. Finally, while the longitudinal tracking across 3 months offered valuable insights into microbial resilience, a longer observational window is required to determine the precise point at which baseline clinical dysbiosis fully resets or stabilizes post-therapy. The results suggest that while the biological trend is evident, a larger sample size might be required to overcome the high natural variance of the human microbiome and reach statistical significance. Further study should consider similar age groups, dietary data, hormonal data, and xerostomia data for a comprehensive analysis.

## 5. Conclusions

This study provides a comprehensive 16S rRNA NGS profile of the oral microbiome across the health-to-periodontitis spectrum within Indonesian demographic. Our findings confirm that alpha diversity indices were notably higher in the gingivitis and periodontitis groups compared to health group, indicating a trend toward increased community richness and evenness in diseases states. Beta diversity findings displayed differences in community structure. While periodontal therapy successfully induces an immediate shift trend toward a health-associated microbial composition, this state is inherently unstable. Ultimately, suppressing bacterial dysbiosis and regulating periodontal homeostasis must be the dual priorities of modern periodontal management in Indonesia. Future investigations should transition from this pilot framework into multi-center, large-scale longitudinal cohorts that encompass a balanced distribution of both male and female participants across diverse age groups and regions. Incorporating multi-omic approaches such as combining full-length 16S rRNA meta taxonomics with meta transcriptomics will be essential to validate whether these observed taxonomic shifts translate directly to functional metabolic changes in the subgingival pocket. Such expanded studies will be vital for developing targeted, population-specific preventive measures and predictive biomonitoring tools for periodontal disease.

## Figures and Tables

**Table 1:** Comparison of alpha diversity between health, gingivitis, and periodontitis.

Alpha Diversity Index	*p*-Value (Kruskall Wallis)	Effect Size (η^2^)	Interpretation
Chao1	0.155	0.192	Large effect
Shannon	0.058	0.410	Large effect
Simpson	0.078	0.346	Large effect

**Table 2:** Comparison of alpha diversity between baseline, 1-month, and 3-month visits in the periodontitis group.

Alpha Diversity Index	*p*-Value (Friedman)	Kendall’s W	Interpretation
Chao1	0.779	0.063	Small effect
Shannon	0.472	0.188	Small effect
Simpson	0.368	0.250	Small effect

**Table 3:** Analysis of top 5 subgingival microbiota at the genus level in health, gingivitis, and periodontitis subjects.

Genus	Periodontal Status			Kruskal-Wallis
	Health	Gingivitis	Periodontitis	*p*-Value
*Neisseria*	0.86 (2.94)	6.65 (11.70)	5.82 (15.44)	0.246
*Stutzerimonas*	3.05 (19.87)	2.12 (3.54)	0.12 (0.35)	0.077
*Capnocytophaga*	18.63 (23.87)	8.88 (7.83)	4.17 (6.27)	0.397
*Veillonella*	0.76 (13.00)	0.24 (7.85)	2.79 (1.84)	0.874
*Prevotella*	0.53 (1.86)	5.90 (3.08)	12.18 (24.02)	0.155

Note: Variables are presented as median (IQR).

**Table 4:** Analysis of top 5 subgingival microbiota at the species level in health, gingivitis, and periodontitis subjects.

Species	Periodontal Status			Kruskal-Wallis
	Health	Gingivitis	Periodontitis	*p*-Value
*Selenomonas noxia*	0.60 (1.51)	2.12 (3.94)	2.55 (1.69)	0.491
*Prevotella oris*	0.18 (1.70)	0.19 (0.49)	0.26 (5.60)	0.981
*Campylobacter gracilis*	4.56 (3.03)	1.34 (1.21)	1.32 (1.26)	0.236
*Capnocytophaga granulosa*	11.02 (14.74)	5.52 (5.97)	1.37 (1.83)	0.087
*Veillonella parvula*	0.20 (4.72)	0.09 (3.43)	1.00 (0.82)	0.874

Note: Variables are presented as median (IQR).

**Table 5:** Analysis of top 5 composition of subgingival microbiota at the genus level in periodontitis (baseline, 1-month, and 3-month).

Genus	Periodontitis			Friedman
	Baseline	1 Month	3 Months	*p*-Value
*Porphyromonas*	5.45 (13.33)	6.58 (11.36)	6.33 (6.03)	0.472
*Prevotella*	12. 18 (24.02)	13.86 (7.43)	2.74 (10.61)	0.472
*Capnocytophaga*	4.17 (6.27)	3.57 (3.85)	8.08 (9.13)	0.105
*Neisseria*	5.82 (15.45)	1.55 (7.49)	3.46 (7.06)	0.472
*Selenomonas*	5.95 (5.13)	5.39 (7.14)	3.87 (2.53)	0.174

Note: Variables are presented as median (IQR).

**Table 6:** Analysis of top 5 composition of subgingival microbiota at the species level in periodontitis (baseline, 1-month, and 3-month).

Species	Periodontitis			Friedman
	Baseline	*p*-Value	3 Months	*p*-Value
*Porphyromonas gingivalis*	2.63 (5.41)	1.08 (3.97)	3.50 (7.00)	1.000
*Porphyromonas endodontalis*	0.67 (4.22)	1.81 (4.61)	0.35 (0.98)	0.368
*Prevotella intermedia*	1.29 (1.18)	3.48 (9.13)	1.30 (1.81)	0.799
*Hoylesella loescheii*	2.66 (2.97)	2.51 (3.51)	2.38 (1.25)	0.472
*Capnocytophaga granulosa*	1.37 (1.83)	1.50 (2.22)	3.22 (5.15)	0.174

Note: Variables are presented as median (IQR).

## Data Availability

The original contributions presented in this study are included in the article. Further inquiries can be directed to the corresponding author.
